# Real World Interstitial Glucose Profiles of a Large Cohort of Physically Active Men and Women

**DOI:** 10.3390/s24030744

**Published:** 2024-01-24

**Authors:** Kristina Skroce, Andrea Zignoli, Federico Y. Fontana, Felipe M. Maturana, David Lipman, Andrea Tryfonos, Michael C. Riddell, Howard C. Zisser

**Affiliations:** 1 Faculty of Medicine, University of Rijeka, 51000 Rijeka, Croatia; 2 Supersapiens Inc., Atlanta, GA 30318, USA; andrea.zignoli@unitn.it (A.Z.); howard@supersapiens.com (H.C.Z.); 3 Department of Industrial Engineering, University of Trento, 38123 Trento, Italy; 4 Department of Diabetes, Endocrinology, Nutritional Medicine and Metabolism (UDEM), Bern University Hospital, University of Bern, 3010 Bern, Switzerland; fdr.fontana@gmail.com; 5 Sports Medicine Department, University Hospital of Tübingen, 72076 Tübingen, Germany; 6 Department of Laboratory Medicine, Division of Clinical Physiology, Karolinska Institute, 171 77 Stockholm, Sweden; a.tryfonos@euc.ac.cy; 7 School of Science, Department of Life Science, European University Cyprus, Nicosia 1516, Cyprus; 8 School of Kinesiology and Health Science, Muscle Health Research Centre, York University, Toronto, ON M3J 1P3, Canada; mriddell@yorku.ca

**Keywords:** gender differences, glycemia, meals, nutrition, exercise, sleep, hypoglycemia, hyperglycemia

## Abstract

The use of continuous glucose monitors (CGMs) in individuals living without diabetes is increasing. The purpose of this study was to profile various CGM metrics around nutritional intake, sleep and exercise in a large cohort of physically active men and women living without any known metabolic disease diagnosis to better understand the normative glycemic response to these common stimuli. A total of 12,504 physically active adults (age 40 ± 11 years, BMI 23.8 ± 3.6 kg/m^2^; 23% self-identified as women) wore a real-time CGM (Abbott Libre Sense Sport Glucose Biosensor, Abbott, USA) and used a smartphone application (Supersapiens Inc., Atlanta, GA, USA) to log meals, sleep and exercise activities. A total of >1 M exercise events and 274,344 meal events were analyzed. A majority of participants (85%) presented an overall (24 h) average glucose profile between 90 and 110 mg/dL, with the highest glucose levels associated with meals and exercise and the lowest glucose levels associated with sleep. Men had higher mean 24 h glucose levels than women (24 h—men: 100 ± 11 mg/dL, women: 96 ± 10 mg/dL). During exercise, the % time above >140 mg/dL was 10.3 ± 16.7%, while the % time <70 mg/dL was 11.9 ± 11.6%, with the remaining % within the so-called glycemic tight target range (70–140 mg/dL). Average glycemia was also lower for females during exercise and sleep events (*p* < 0.001). Overall, we see small differences in glucose trends during activity and sleep in females as compared to males and higher levels of both TAR and TBR when these active individuals are undertaking or competing in endurance exercise training and/or competitive events.

## 1. Introduction

Circulating glucose is a key energy source for working muscles and the central nervous system during exercise [1]. Assuming that a “normal” mean blood glucose concentration is between ~3.9 and 7.0 mmol/L (70–126 mg/dL) for the average adult, this equates to only about 4 g (i.e., one teaspoon) of glucose in the blood stream and about an additional ~10 g of glucose in the interstitial fluid at any point in time during rest [2]. Several mechanisms are at play to try to preserve this level during exercise in healthy individuals, like neuroendocrine responses that involve both feed-forward and feedback components as well as a complicated set of endocrine responses involving the sympathetic nervous system and its capabilities for direct and endocrine signaling, initiating cardiopulmonary, cardiovascular and other fight or flight autonomic responses [1]. However, it has long been established that some forms of endurance exercise, such as marathon running, can result in precipitous drops in glucose concentrations and even severe hypoglycemia [3]. While hypoglycemia can be avoided with carbohydrate feeding during exercise, which also delays fatigue and enhances performance [4], the optimal timing and dose of carbohydrate ingestion remains under some debate [5]. In fact, athlete practices surrounding intake often do not match contemporary sports nutrition guidelines [6], which complicates the field of sports nutrition even more.

A continuous glucose monitor (CGM) is a wearable biosensor that automatically and repeatedly measures glucose at regular intervals (ranging from every 1 to every 15 min) from the interstitial fluid (ISF) [7]. This wearable technology is minimally invasive (i.e., a small sterile filament is placed under the skin into the ISF) and reasonably cost effective (~$7 USD/day) and provides immediate visibility of the glucose levels measured from ISF on a mobile display (i.e., a smart phone application, wrist-worn wearable and/or cycling computer). Software programs also allow for event tagging (e.g., meals, snacks, sleep, exercise events, etc.) and offer information on glycemic trends over time (daily, weekly, etc.) and proportions of time in various glucose concentrations. Parameters obtained from CGM readings are regularly being used both for daily management of patients with diabetes, and the accuracy of these devices during meals and exercise has been validated in people living with diabetes [8].

In athletes without diabetes, Ishihara and co-authors [9] used CGM as a potential method to suggest optimal carbohydrate intake timing and dosing strategies in ultramarathon runners without diabetes. The authors found that glucose concentration was positively correlated with running speeds in different segments, and energy and carbohydrate intake was also positively correlated with overall running speed. However, to date, reporting of CGM values for athletes without diabetes during training and/or competition has not been widely studied, given that the product has traditionally only been available in populations with diabetes. The commercial use of CGMs in the European marketplace for people without diabetes now allows for the possibility of using this technology in real-world settings that include nutrient intake, rest days, training and competition. Profiling CGM metrics and glucose excursions in otherwise healthy men and women is essential for the interpretation of glucose measurements in apparently metabolically “healthy” individuals and could serve as a reference point for future nutrient and carbohydrate periodization studies, which are recommended to enhance sports performance in active men and woman [10]. As such, the primary aim of this study is to retrospectively characterize, in a real-world setting, various CGM metrics around meals, sleep and exercise in a large group of active healthy individuals to better understand CGM dynamics in active people living without metabolic disease. Overall, we propose that this observational study will serve as a benchmark to help us understand “normative” CGM dynamics in active people living without diabetes.

## 2. Materials and Methods

### 2.1. Study Design and Participants

This is a retrospective, single-arm, observational study. Data are included (user agreement allows for unidentified data to be used for research purposes) from Supersapiens commercial users. Study participants (demographics reported in Results) agreed to anonymously share their CGM data by wearing the Abbott Libre Sense Glucose Sport Biosensor under free-living conditions and logging food, sleep and exercise events using the Supersapiens (www.supersapiens.com, accessed on 1 January 2024, Supersapiens Inc., Atlanta, GA, USA) mobile app. The authors were unable to retrieve users’ identities, as each user was assigned a unique code before exporting the data.

Participants were healthy adult men and women who claim to exercise regularly (from 2 to 7 times a week). Participants were asked to select their “gender,” rather than their biological sex, during application onboarding, so the data provided within are denoted as “male” or “female.” Eligibility criteria for study participants were as follows: age > 18 years, no chronic illness or medications that might affect glucose metabolism, no known allergy to isopropyl alcohol used to disinfect skin or medical grade adhesives and no skin lesions, scarring, redness, infection or edema at the CGM application sites that could interfere with device placement or the accuracy of interstitial analyte measurements. Women also claimed that they were not knowingly pregnant at the time of CGM usage.

The Abbott Libre Sense Sport Glucose Biosensor (Abbott Diabetes Care, Alameda, CA, USA) was worn for up to 14 days (sensor wear duration) under free-living conditions. The biosensor measures interstitial glucose levels within a 55–200 mg/dL range every minute and uses an integrated surface recorder/transmitter that communicates in real time to a smart phone application via Bluetooth^TM^ technology (www.supersapiens.com, accessed on 1 January 2024, Supersapiens Inc., Atlanta, GA, USA). While connected to the CGM, participants were instructed to log their activities in real time, including food intake (i.e., meals, carbohydrate containing beverages, gels and snacks) and exercise, using the smartphone app. Only participants with at least 7 total days (i.e., at least 50% of available data) of CGM data were included in the analysis. The CGM was not blinded, so the user could see their readings in real time.

### 2.2. Data Handling and Statistical Analyses

All glucose outcomes are reported as mean and standard deviation (SD), unless otherwise stated. Each CGM reading is counted as a data point and is summarized in the glucose outcomes at either the participant level or at the event level (24 h, meals, exercise and sleep). Raw CGM data were exported from the Supersapiens database together with the corresponding recorded time stamp in the user’s local time. From these, information regarding the CGM value and the duration of the periods spent in the different glucose level ranges could be computed. Only data collected at a frequency of 4 samples per hour (one sample every 15 min) or higher (maximum one data sample every minute) were considered. When a day presented data gaps, it was excluded by the analysis. The data points have not been filtered but only linearly interpolated. The following glucose outcomes were assessed: (*i*) overall (24 h average data, from midnight to midnight); (*ii*) during sleep (as reported by logged sleep events); (*iii*) during exercise events; and (*iv*) during meal events (0–2 h post meal/snack intake). Individual daily glucose nadirs and peaks were not estimated, since CGMs that report glucose values at high frequency rates (between 1–5 min) are influenced by several potential sources of error, including sensor time lags, sensor biases, a transient loss of sensor precision (i.e., sensor noise) and missed sensor readings [11]. However, the percentage of time spent within specific glucose ranges (i.e., % time below range [TBR, <70 mg/dL or <3.9 mmol/L]; % time in range [TIR, 70–140 mg/dL or 3.9–7.8 mmol/L]; % time above range [TAR, >140 md/dL or >7.8 mmol/L]) were calculated from the CGM readings that fell within that zone divided by the total number of CGM readings from the participant, represented as a percentage [12]. At least two sensor values > 70 mg/dL that were ≥15 min apart with no intervening values < 70 mg/dL were required to end an event considered “below range” [12]. All statistical analyses were performed in RStudio (R Core Team, 2020, https://www.R-project.org/, accessed on 1 January 2024,).

Potential differences in glucose parameters (average, variability) across the 24 h and during exercise, food and sleep events related to BMI categories were assessed using a one-way ANOVA. The significance level was set at *p* < 0.05. Gender-related differences in glucose levels during exercise, food and sleep events as well as 24 h trends were assessed using *t*-tests for independent groups. The significance level was set at *p* < 0.05. To evaluate size and significance of gender, effect sizes were estimated by calculating Cohen’s d values and their corresponding 95% confidence intervals (CI). According to common practice, effects were considered small (≥0.20), moderate (≥0.50) or large (≥0.80) [13].

## 3. Results

Overall, 12,504 adults (age = 40 ± 11 years, BMI = 23.8 ± 3.6 kg/m^2^; 23% self-identified as women) participated in the study. Anthropometric data, as well as self-reported exercise training status and primary training mode and/or sport, are reported in Table 1. According to the self-reported training data, most of the cohort were characterized as intermediate-level “athletes” (33%), meaning 3–4 workouts per week, followed by advanced (31%, 4–5 workouts. per week), beginners (15%, 1–2 workouts per week) and experts (12%, >5 workouts week). A total of 9% of athletes did not report their training status. Approximately 70% of the participants were endurance athletes, with the primary sport of cycling representing the largest training activity of the cohort (27%), followed by triathletes (24%) and runners (19%) (Table 1).

**Figure 1:**
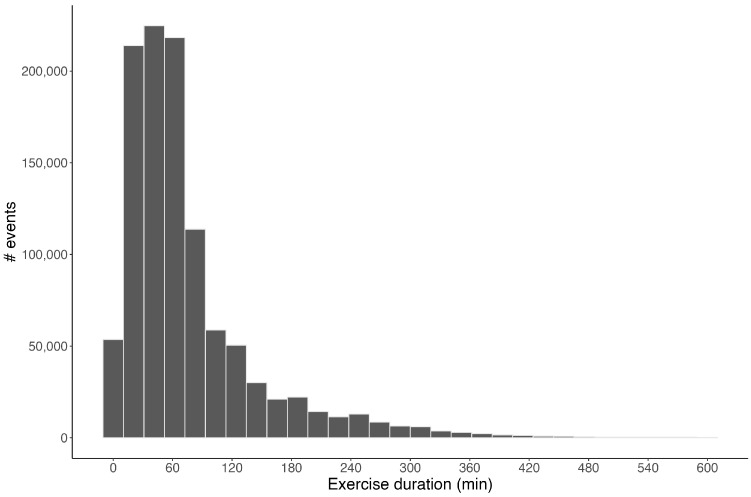
Distribution of exercise duration across all 1,084,824 exercise events logged into the Supersapiens mobile application by 12,504 CGM users. The average exercise event duration logged was 77 ± 75 min.

**Figure 2:**
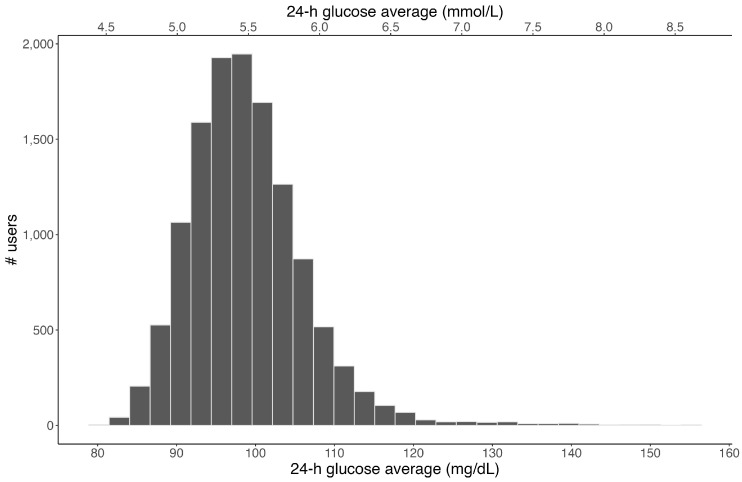
Distribution of the 24 h glucose average across all 12,504 CGM users. Note: Sensor captures interstitial glucose levels within 55–200 mg/dL range only.

**Figure 3:**
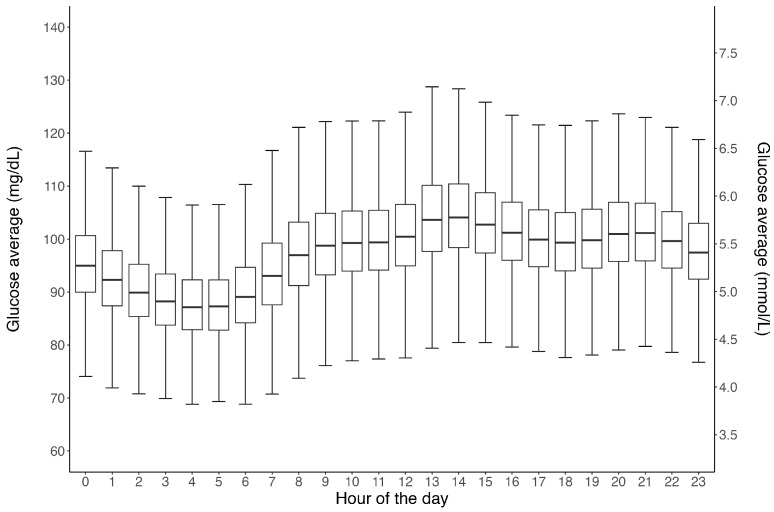
Box and whisker plots showing the 24 h mean glucose data from all 12,504 CGM users. Note: Each sensor wear time is limited to 15 days. Horizontal lines within the boxes represent medians, boxes represent the interquartile range and vertical lines (whiskers) represent the 95% confidence intervals.

In total, 1,084,824 exercise events and 274,344 meal events were created. The distribution of exercise duration across all events is presented in Figure 1, and the average exercise duration itself was 77 ± 75 min. The distribution of the average 24 h glucose concentration across all 12,504 participants is shown in Figure 2. Overall, the mean daily glucose was 97 ± 10.6 mg/dL, ranging from 80–155 mg/dL. Figure 3 shows the distribution of average glucose levels by time of day. In general, the majority of subjects (85%) presented an overall (24 h) glucose average between 90 and 110 mg/dL, with the group nadir appearing at ~04:00 and the group peak at ~13:00.

**Figure 4:**
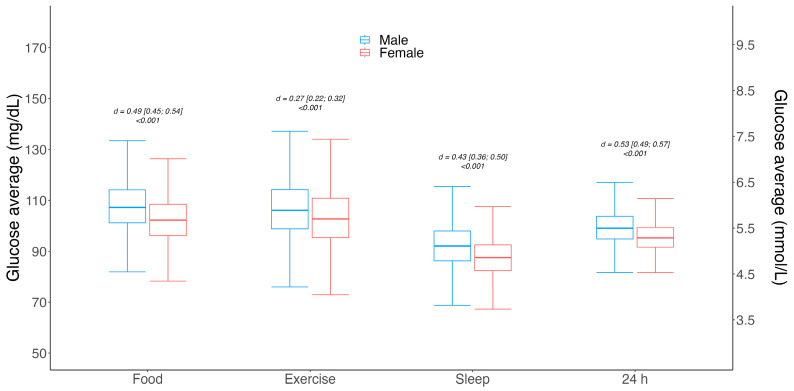
Box and whisker plots for gender-associated mean glucose level differences for meal, exercise and sleep events, as well as overall 24 h. Gender differences were assessed using Student’s *t*-test for independent groups, and significance was set to *p* < 0.05. Effect sizes are shown as Cohen’s d values and their corresponding 95% confidence intervals (CI). Horizontal lines within the boxes represent medians, boxes represent the interquartile range and vertical lines (whiskers) represent the 95% confidence intervals for males (blue) and females (red).

The 24 h glucose average and variability between males and females, as well as glucose levels during different logged events (i.e., meals, exercise and sleep), are shown in Figure 4. Different event-level glucose averages were observed between genders; males had slightly higher glucose values compared to females during meals (107 ± 16 mg/dL vs. 102 ± 15 mg/dL | d = 0.49 | [95% CI 0.45; 0.54] | *p* < 0.001); during exercise (107 ± 13 mg/dL vs. 104 ± 12 mg/dL | d = 0.27 | [95% CI 0.22; 0.32] | *p* < 0.001); and in overall 24 h glycemia (100 ± 11 mg/dL vs. 96 ± 10 mg/dL | d = 0.53 | [95% CI 0.49; 0.57] | *p* < 0.001).

The distribution of the overall 24 h glucose average and variability among BMI categories and self-reported training level is reported in Table 2. Similarly, glucose average and variability reported during exercise events, meals and sleep across BMI categories and self-reported training level are presented in Table 2. No meaningful differences in glucose parameters were detected among the four distinct BMI categories.

**Figure 5:**
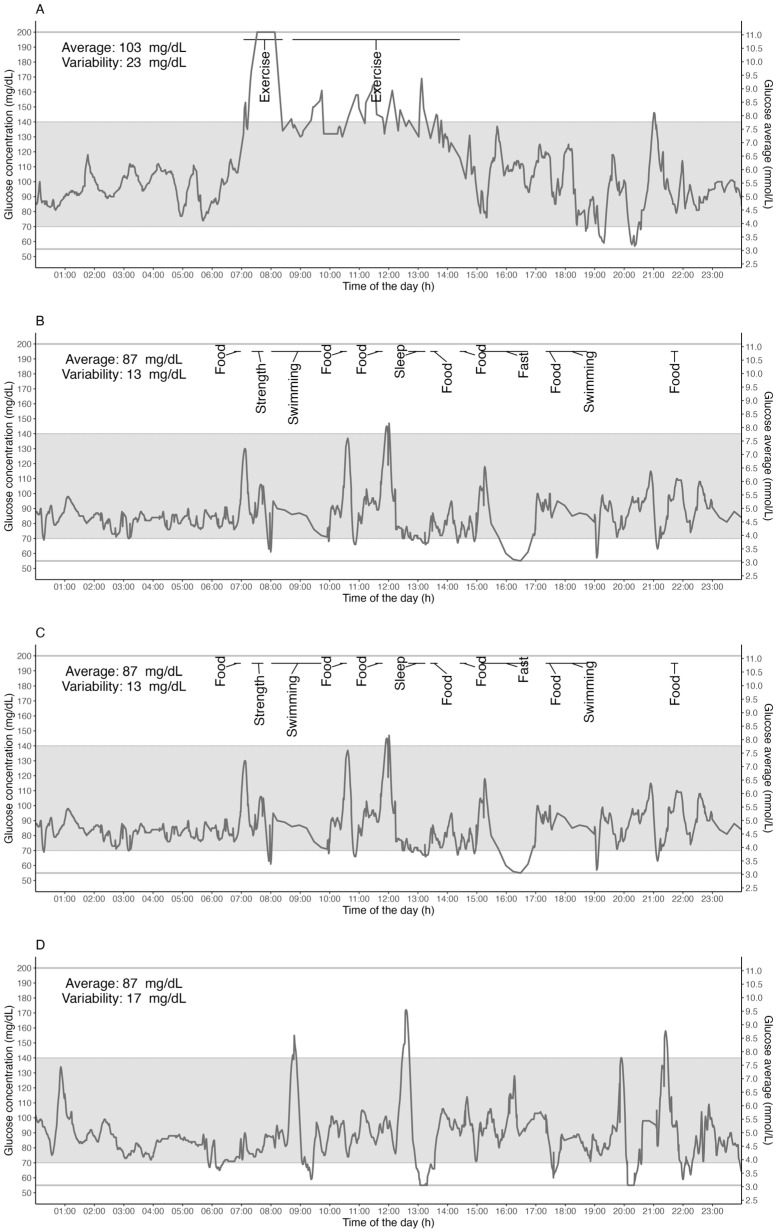
Individual traces give detailed insight into possible individual variations. Examples of days for representative users were chosen from the database, and they delineate 24 h glucose response on a long-race day ((**A**) a well-trained long-distance trail female runner in a race); a double-training day ((**B**) an elite male swimmer); a single-training day ((**C**) an elite female swimmer); and a rest day ((**D**) well-trained long-distance male trail runner). Gray horizontal lines represent the limits of the lowest and highest measurable glucose concentrations (i.e., 55 mg/dL and 200 mg/dL). The gray band area represents the 70–140 mg/dL range of concentration.

Individual example traces shown in Figure 5 provide more detailed insight into the inter-individual variability in glycemia across a 24 h period with representative activity and sleep data. Different types of users were chosen as representative examples from the database, representing a 24 h glucose response to an ultra-endurance race activity (Figure 5A: a well-trained long-distance female trail runner in a race); a double-training day (Figure 5B: an elite male swimmer); a single-training day (Figure 5C: an elite female swimmer); and a rest day (Figure 5D: well-trained male long-distance trail runner). Note: Sensor captures interstitial glucose levels within the 55–200 mg/dL range only.

### 3.1. Time below Range; TBR (<70 mg/dL)

Across the 24 h, 3.4% of time was spent with glucose levels <70 mg/dL, which is considered as the percentage of time below the glycemic target range (%TBR), with an average of 5 ± 3 occurrences per day and an average duration of 11 ± 21 min per occurrence. Females spent on average 0.8% more TBR when compared to males (4.0% vs. 3.2% | d = 0.32 | [95% CI 0.28; 0.36] | *p* < 0.001). They also had slightly longer time with each event relative to males (11 min vs. 10 min | d = 0.21 | [95% CI 0.17; 0.25] | *p* < 0.001) for data collapsed across all meals, sleep and exercise events. During exercise, the mean TBR was 11.9 ± 11.6%, lasting an average duration of 8.3 ± 11.3 min per occurrence, with an average of 1.7 ± 1.4 occurrences per exercise activity. No differences were detected between males and females for %TBR during exercise (all d_s_ < 0.1 | all *p*_s_ > 0.05). During sleep, TBR averaged 3.8 ± 6.6% in the cohort, with occurrences averaging 3.2 ± 2.2 times per night and an average duration of 14 ± 15 min per occurrence. Females had slightly more TBR than males during sleep (3.2 ± 5.8% vs. 3.0 ± 5.0% | d = 0.12 | [95% CI 0.04; 0.20] | *p* = 0.002), which translated to greater total duration in the TBR for females vs. males (15 ± 23 min vs. 13.3 ± 19.7 min | d = 0.28 | [95% CI 0.20; 0.36] | *p* < 0.001).

### 3.2. Time above Range: TAR (>140 mg/dL)

Across the 24 h, 3.6 ± 3.9% of time was spent >140 mg/dL, which is considered the percentage of time above the tight glycemic range (TAR). TAR had an average of 3.6 ± 2.2 daily occurrences and an average duration of each occurrence lasting 51.5 ± 57 min. Women spent, on average, 1% less time in TAR than men (2.8 ± 2.9% vs. 3.8 ± 4.2% | d= −0.26 | [95% CI −0.29; −0.22] | *p* < 0.001) with a shorter average event duration (41.2 ± 42.8 min vs. 54.6 ± 60.5 min | d = −0.05 | [95% CI −0.09; −0.01] | *p* = 0.01). During exercise, TAR represented 10.3 ± 16.7% of the total time with an average duration of 11.5 ± 17.4 min and, on average, 2.3 ± 2.2 occurrences of TAR per exercise event. No statistical differences were detected between genders with regard to the duration of each TAR occurrence during exercise (11.3 ± 17 vs. 11.5 ± 17.5 min | d= −0.06 | [95% CI −0.11; −0.01] | *p* = 0.05). During sleep, participants spent 2.9 ± 6% TAR, 1.8 ± 1.3 times per night, with an average duration of each occurrence of 11 ± 19 min. Gender did not appear to influence TAR sleep metrics (all d_s_ < 0.1 | all *p*_s_ > 0.05).

## 4. Discussion

To the best of our knowledge, this is the largest CGM study to date that focuses on 24 h CGM metrics for exercise and meals in healthy and physically active adults. We included 12,504 physically active participants, who ranged from recreationally active to elite level athletes, and provide various CGM metrics that help establish new gender-specific TIR, TAR and TBR profiles as they relate to exercise and sleep. Overall, men had slightly higher glucose values compared to women during meals and in overall 24 h glycemia.

This large cohort study of active males and females complements other cohort studies of CGM metrics in the general population [14,15,16,17,18] but is the first to focus on glycemia during sleep and endurance exercise. Unlike these other smaller studies, these data reveal small differences between genders in overall 24 h glycemia, with women having slightly lower 24 h mean glucose levels as well as lower glucose levels in response to meals and sleep than men. While TBR events were reasonably frequent in both men and women, averaging five occurrences per day with about 3.4% TBR, the duration was typically brief, (i.e., ~8 min per exercise-related occurrence vs. 11 min in the overnight period), and the gender differences could be deemed small (i.e., only 0.8% more TBR, which is equivalent to an extra 11 min per day < 70 mg/dL). Overall, the active males and females spent most of the time in the range of 70–140 mg/dL, as previously reported by D’Souza et al. [18]. Taken together, these findings are in line with the notion that healthy individuals with intact glucose counterregulatory hormones have very little sustained exposure to biochemical hypoglycemia (glucose < 70 mg/dL) [19].

Importantly, despite a very active population in our cohort, and therefore assumingly a cohort with higher daily carbohydrate intake, with frequent bouts of highly variable exercise intensity, the overall 24 h glucose levels were largely within the expected normative range. Nonetheless, frequent small deviations from the normative range, and, in particular, in time above range, during exercise were observed. This study is the first to demonstrate that real-world exercise can be associated with sustained elevations in glycemia, perhaps as a mechanism to help support fuel delivery to the working muscles. We envision that these novel observations may improve our understanding of the normative glucose response to meals, exercise and sleep in active men and women without diabetes.

In this dataset, we noted several frequent periods of TBR during prolonged endurance exercise. However, we also noted that the duration of each TBR event lasted only a few minutes on average (i.e., lasting 8 ± 11 min per event), and episodes of sustained hypoglycemia were rare. As such, we postulate that these brief periods of apparent “in-exercise hypoglycemia” might be reflective of something termed exercise-associated rebound hypoglycemia or reactive hypoglycemia, which can occur if carbohydrate feeding is initiated before physical activity, thereby resulting in a transient increase in insulin secretion that can result in hypoglycemia once the exercise begins and glucose disposal rates rise [20,21,22,23]. This is a transient condition that can be caused by the timing of the pre-exercise carbohydrate intake [24]. Our findings also support the notion that the body has a clear glucose counterregulatory defense mechanism to minimize hypoglycemia exposure during exercise [1,2]. Recent work from a similar dataset suggests that reactive hypoglycemia is detectable in about 8% of all endurance exercise events, with only about 15% of all individuals experiencing hypoglycemia in >20% of their events [25]. Based on our analyses, we propose that reactive hypoglycemia is more likely to occur with pre-exercise food timing between ~30 and ~90 min, with a peak risk if carbohydrates are consumed 60 min before exercise. However, more controlled studies are needed to define the exercise type and intensity that increase rebound hypoglycemia risk and its relationship to the quantity and quality of the nutritional intake before the activity, including the macronutrient mix and carbohydrate type.

**Carbohydrate intake and TAR**

In order to maintain homeostasis, the human body is dependent on tight control of its circulating blood glucose levels [2]. This dataset of self-reported healthy subjects shows that the overall 24 h interstitial glucose level was indeed maintained at 99 ± 7 mg/dL. This finding is in line with recent observations of Shah et al. [26] in 153 apparently healthy (i.e., non-diabetic subjects) adults and youth where the mean 24 h glucose was 98–99 mg/dL for those less than 60 years of age. It is important to emphasize that in our current study, all subjects were highly active participants, including some elite endurance athletes who traditionally have very high carbohydrate intakes during exercise (60–90 g of carbohydrates per hour) and also tend to have high-carbohydrate snacks and meals before and after training events [27]. It is typical that athletes consume high amounts of simple carbohydrates for performance reasons [28], often relying on high glycemic index sport beverages and liquids before, during and after competitive exercise events [29]. Despite these assumingly high intakes of daily carbohydrates, we failed to observe significant patterns of hyperglycemia in the events logged during the day or night in this cohort. However, we did note that the % TAR was unusually high during exercise (>10%), which may be suggestive of modest overfueling with carbohydrates and/or exercise-associated increases in glycemia because of increased counterregulatory hormones [30,31]. This finding is in contrast to the previously mentioned cohort of non-athletes where glucose levels dropped by ~15 mg/dL during physical activity/exercise in an ancillary study [14]. In our more active cohort, we clearly observed prolonged periods of sustained TAR during exercise, with a mean duration of 10.3 ± 16.7% per exercise event. This finding is of particular interest to athletes, as it proves that despite the possible in-exercise periods of elevated glycemia during periods of activity and with assumed high levels of carbohydrate intake overall, they can still sustain an overall high %TIR.

**Variations caused by sleep**

Given the associations between high glucose variability and long-term vascular damage [32], it is important to note that the glucose variability of our cohort is not higher than previous observations in healthy subjects [14,26]. However, we also noted in this study cohort that glucose levels during sleep appeared to show a circadian variation, with CGM values being lower during the night and gradually dropping to a nadir in the morning before the typical waking hour (nadir was at 4 A.M., while sleep often ended 2–3 h later). In a very limited number of studies specifically focused on overnight glucose levels in subjects without diabetes [33,34,35], a small increase in insulin concentrations and/or secretion in the face of stable [35] or minimally elevated [33,34] glucose levels has been observed toward the end of the nocturnal sleep period. These findings have been interpreted as indicating an increased need for insulin secretion in the early morning hours to combat the circadian rise in whole-body insulin resistance in the early morning, perhaps contributed to by an early morning rise in cortisol [36]. While the underlying mechanisms are not clearly defined for this cohort, it is possible that a similar dawn phenomenon (i.e., an increase in blood glucose levels and/or insulin requirements in the pre-breakfast hours) occurs in insulin-sensitive athletes similar to that occurring in the general population, albeit with considerable inter-individual variation [37,38].

**Training status and gender-related differences**

Interestingly, glucose levels during exercise appeared to be about 10 mg/dL higher during exercise in athletes that self-identified as exercise/endurance “experts” when registering in the app (Table 2). Nonetheless, this may be either due to higher relative intensity workouts or due to higher carbohydrate feeding rates prior to or during the exercise sessions. Future research is needed to determine if an ideal glycemic range exists for endurance competition and/or performance in healthy athletes and if CGM can be used to target this range by further optimizing any already existing evidence-based nutritional intake strategies for performance [39].

In contrast to the lack of significant differences reported by Zhou et al. in 2009 [40], our findings suggest that men tend to have significantly higher glucose levels than women across the 24 h, with no differences in glucose variability. Lower glucose concentrations in women, as compared to men, were maintained in response to meal and sleep events, but the apparent gender-related differences tended to diminish during exercise. Our larger sample size, resultant greater capacity to detect smaller gender-related differences and study population being highly active may also be potential reasons for the differences observed. In fact, it was previously shown that when subjects were matched for physical fitness and during a hyperinsulinemic-euglycemic clamp, insulin sensitivity was greater in women as a result of higher glucose disposal by skeletal muscles [41]. Furthermore, sex differences have been reported in skeletal muscle, with a higher proportion of type I fibers and capillary density in women, of which both may favor enhanced insulin action [42]. This is true regardless of the fact that women typically have lower skeletal muscle mass, higher adipose tissue mass, higher circulating free fatty acids (FFA) and higher intramyocellular lipid content [38,43]. Unfortunately, we did not capture menstrual phase status for the women in this study. Nonetheless, a recent study found apparent differences in glucose homeostasis during luteal vs. follicular phases of the menstrual cycle [44], which may help explain the apparent gender-related differences in our study. Future research should focus on the effects of menstrual status and menstrual phase on various CGM metrics during different forms of exercise in women.

To the best of our knowledge, this is the largest cohort studied to date of physically active men and women wearing CGM over a prolonged wear time while regularly engaging in endurance exercise events in a free-living setting. However, given the observational nature of the study, a number of limitations should be considered. Data regarding meals and exercise were from smart-phone-enabled diary entries made by participants; therefore, it is possible that not all meals, snacks, or exercise were accurately recorded. Moreover, the confounding effect of unreported meals or snacks consumed before and/or during exercise on glucose levels cannot be ruled out. Other than gender, age on its own may also affect glycemic responses, particularly in adults over the age of 60 years [26,40], and our dataset was limited to mostly young and active participants (i.e., only 3% of our participants were >60 years old). Finally, we did not capture several other potentially important exercise parameters, such as relative exercise intensity, which likely impact glucose responses. As such, further studies are required to examine the effects of different exercise sessions on glucose excursions.

## 5. Conclusions

This study presents a comprehensive characterization of CGM measures on a large non-diabetic population in a real setting and everyday life. Despite the obvious benefits of CGM use in the diabetic population, athletes and the general population may also benefit from continuous glucose readings, as they provide live visibility of glucose fluctuations during a variety of everyday life situations. We believe that the results we presented here may assist future research involving CGM data and hope that our study will inspire additional initiatives to expand this knowledge base in the future.

## Figures and Tables

**Table 1:** Participant’s anthropometric data, self-reported exercise training status and primary training sport.

**Gender**	
Female	23% (n = 2872)
Male	77% (n = 9632)
**Age**	
<20 years	2% (n = 210)
20–40 years	54% (n = 6704)
40–60 years	41% (n = 5179)
>60 years	3% (n = 411)
**BMI**	
Underweight (<18.5)	2% (n = 299)
Normal (18.5–25)	65% (n = 8135)
Overweight (25–30)	22% (n = 2720)
Obesity (>30)	11% (n = 1350)
**Fitness level**	
Not Reported	9% (n = 1118)
Beginner (1–2 workouts/week)	15% (n = 1819)
Intermediate (3–4 workouts/week)	33% (n = 4147)
Advanced (4–5 workouts/week)	31% (n = 3914)
Expert (>5 workouts/week)	12% (n = 1506)
**Primary sport**	
Other	10% (n = 1272)
Not Reported	5% (n = 588)
Triathlon	24% (n = 3033)
Cycling	27% (n = 3342)
Running	19% (n = 2339)
Swimming	1% (n = 146)
Crossfit	4% (n = 516)
Obstacle Course Racing	1% (n = 66)
Gym	9% (n = 1097)
Football (Soccer)	1% (n = 105)

**Table 2:** Average glucose levels during meal, exercise and sleep events, as well as 24 h glucose averages for different BMI and training level groups.

	Meals	Exercise	Sleep	24 h
**Age**				
<20 years	106 ± 12 (94–120)	112 ± 13 (98–128)	92 ± 10 (82–104)	99 ± 8 (90–108)
20–40 years	106 ± 10 (93–119)	107 ± 13 (92–123)	91 ± 10 (79–102)	98 ± 7 (90–106)
40–60 years	108 ± 12 (95–121)	106 ± 13 (92–122)	93 ± 11 (81–105)	100 ± 8 (91–109)
>60 years	113 ± 14 (98–132)	108 ± 15 (90–125)	96 ± 13 (85–109)	104 ± 12 (93–118)
**BMI**				
Underweight (<18.5)	106 ± 10 (95–121)	106 ± 12 (92–120)	91 ± 10 (80–104)	98 ± 7 (89–107)
Normal (18.5–25)	107 ± 11 (95–120)	107 ± 13 (93–123)	91 ± 10 (80–104)	99 ± 7 (91–107)
Overweight (25–30)	106 ± 11 (93–120)	105 ± 13 (90–121)	92 ± 11 (81–105)	99 ± 8 (90–108)
Obesity (>30)	106 ± 13 (92–121)	104 ± 14 (89–122)	91 ± 13 (78–103)	99 ± 9 (90–109)
**Fitness level**				
Beginner	106 ± 13 (92–120)	102 ± 14 (87–118)	91 ± 12 (79–106)	99 ± 9 (89–108)
Intermediate	106 ± 11 (94–120)	105 ± 13 (91–121)	91 ± 11 (79–103)	98 ± 7 (90–107)
Advanced	108 ± 10 (95–120)	107 ± 12 (94–122)	92 ± 10 (82–104)	99 ± 7 (91–108)
Expert	108 ± 10 (96–121)	112 ± 12 (98–128)	93 ± 10 (82–104)	100 ± 7 (92–109)
Not Reported	107 ± 12 (93–121)	107 ± 15 (90–125)	92 ± 12 (80–105)	99 ± 9 (90–110)

Data are expressed as mean value ± SD. The 10th and 90th percentiles of the values are also reported in the brackets.

## Data Availability

The datasets presented in this article are not readily available because the data are part of Supersapiens Inc. commercial user’s database. Requests to access the datasets should be directed to the corresponding author.
